# TMS-EEG indexes abnormal GABAergic signalling in patients with schizophrenia

**DOI:** 10.1192/bjo.2021.185

**Published:** 2021-06-18

**Authors:** Sukhwinder S Shergill, Viviana Santoro, Lorenzo Rocchi, Meng Di Hou, Isabella Premoli

**Affiliations:** 1 Department of Psychosis Studies, Institute of Psychiatry, Psychology and Neuroscience (IoPPN), King's College London; 2 Department of Basic and Clinical Neuroscience, Institute of Psychiatry, Psychology and Neuroscience, King's College London; 3 Department of Clinical and Movement Neurosciences UCL Queen Square Institute of Neurology, University College London

## Abstract

**Aims:**

Transcranial magnetic stimulation (TMS) is a non-invasive brain stimulation tool designed to probe the strength of inhibitory and excitatory neurotransmission in the cortex. Combined with electromyography, paired-pulse TMS paradigms have revealed a deficit in inhibition mediated by GABA-A receptors in patients with schizophrenia. Combined TMS-electroencephalography (TMS-EEG) provides a more detailed examination of cortical excitability and may shed more light into the pathophysiology of schizophrenia. Of the various peaks of the TMS-evoked EEG signal, responses at 45 (N45) and 100 ms (N100) likely reflect GABA-A and GABA-B receptor-mediated inhibition, respectively. Responses at 25 ms (P25) are affected by voltage-gated channel ligands, whereas glutamatergic processes may be related to the P70 component. We here aim to systematically investigate the role of these neural processes in patients with schizophrenia by using TMS-EEG.

**Method:**

TMS-evoked EEG potentials (TEPs) were recorded in patients with schizophrenia (n = 19) and in age-matched healthy controls (n = 17). 150 TMS pulses at 90% of resting motor threshold were applied over the left primary motor cortex during EEG recording. Differences in TEPs between the two groups were analysed for all electrodes and for time windows corresponding to each TEP (P25: 0.015-0.035 ms; N45: 0.035-0.06 ms; P70: 0.035-0.06 ms; N100: 0.09-0.14ms) by applying multiple independent sample t-tests. Further, a cluster-based permutation analysis approach was implemented to correct for multiple comparisons.

**Result:**

Compared to controls, patients showed amplitude reduction for the P25 (negative and positive cluster; p < 0.001 and p = 0.04, respectively), N45 (negative and positive cluster; p < 0.001 and p = 0.001, respectively) and P70 component (negative and positive cluster; p = 0.04 and p = 0.004, respectively).

**Conclusion:**

There results extend on previous literature about impairment of GABA-A receptor mediated inhibition in schizophrenia, as demonstrated by the N45 amplitude reduction, whereas no significant differences in GABA-B index (i.e., N100) were revealed. Our results also showed that, although specific mechanisms underlying P25 and P70 have not been fully elucidated yet, excitatory neurotransmission is altered in this clinical population. To conclude, TMS-EEG may provide a more comprehensive view of the inhibitory and excitatory mechanisms involved in the pathophysiology of schizophrenia.

